# Multitargeted Modulation of Skin Dullness by HNHT Formulation: Synergistic Inhibition of Melanogenesis, Glycation, and Lipofuscin Deposition

**DOI:** 10.1111/jocd.70789

**Published:** 2026-05-18

**Authors:** Zheng Wang, Yumei Fan, Peixue Ling

**Affiliations:** ^1^ Meyer Bio‐Medicine Co., Ltd. Jinan China; ^2^ Shandong Meimao Pharmaceutical Co., Ltd. Jinan China; ^3^ National Glycoengineering Research Center Shandong University Jinan China

**Keywords:** anti‐glycation, HNHT, lipofuscin, melanin, skin dullness

## Abstract

**Background:**

Skin dullness, characterized by pigmentation disorders and yellowing, is a major cosmetic concern in Asian populations. Lipofuscin exhibits ultraviolet (UV)/visible light spectral absorption, reduces skin reflectance, and contributes to dullness, whereas existing therapies targeting melanogenesis and glycation lack comprehensive efficacy. This study aimed to evaluate the multimodal anti‐dullness effects of a novel formulation (HNHT: hyaluronic acid, niacinamide, hydrolyzed red algae, and tranexamic acid).

**Methods:**

In vitro models (melanocytes, keratinocytes, fibroblasts, ex vivo skin model, and three‐dimensional (3D) skin models) were used to assess melanin regulation, glycation inhibition, and lipofuscin clearance.

**Results:**

In ultraviolet B (UVB)‐exposed 3D skin models, 12.5% HNHT significantly improved skin color, appearance, and brightness (8.44% increase in L* value) and reduced melanin content (12.5% inhibition). Then, 12.5% HNHT downregulated the expression of key melanogenic genes (*TYR* and *MITF*; *p* < 0.001) in melanocytes and decreased UVB‐induced α‐melanocyte‐stimulating hormone secretion in keratinocytes (2867.78 versus 3444.72 pg/mL; *p* < 0.05). The formulation enhanced melanosome clearance through autophagy activation (174% LC3‐II increase, 80% GP100 reduction; *p* < 0.01) and boosted antioxidant capacity via Nrf2 activation and reactive oxygen species reduction (*p* < 0.05). In glycation, 50% HNHT upregulated anti‐glycation enzymes (*GLO1*, *GLO2*, and *GPX1*) and reduced carboxymethyllysine (CML) by 23.16% in fibroblasts (*p* < 0.01), while 100% HNHT decreased CML by 34.93% in ex vivo skin model. Furthermore, 50% HNHT suppressed hydrogen peroxide‐induced lipofuscin accumulation by 52.20% (*p* < 0.01).

**Conclusions:**

These findings highlight HNHT as a multitargeted therapeutic agent that alleviates skin dullness through melanogenesis inhibition, enhances melanosome degradation, suppresses glycation, mitigates oxidative stress, and promotes lipofuscin clearance, thereby establishing a novel therapeutic paradigm against solar radiation‐induced hyperpigmentation and skin yellowing.

## 1 Introduction

Skin dullness is a prevalent concern among Asian women and is mainly characterized by unhealthy pigmentation and a lack of radiance or uneven texture [1]. Pigmentation and skin yellowing are the principal causes of skin dullness are pigmentation and skin yellowing resulting from aging [1, 2]. Rigal et al. [3] investigated skin color changes with age across four ethnic groups and observed that, while all groups experienced significant skin darkening, Chinese individuals exhibited more pronounced yellowing. Skin yellowness may result from carbonyl or glycosylated protein accumulation during cellular bio‐oxidation and lipofuscin deposition [2, 4, 5]. Therefore, to improve skin dullness, it is essential to address multiple factors, including inhibiting melanin deposition, reducing protein carbonylation, glycation modifications, and lipofuscin accumulation.

Pigmentation is closely associated with the presence of melanin and lipofuscin. Melanin, a darkly pigmented biopolymer, is synthesized by lysosome‐like subcellular organelles called melanosomes present in melanocytes. Keratinocyte‐derived α‐melanocyte‐stimulating hormone (α‐MSH) activates melanocortin 1 receptor (MC1R) on melanocyte membranes, triggering downstream signaling cascades that involve microphthalmia‐associated transcription factor (MITF) activation. As the master regulator of melanogenesis, MITF directly regulates the tyrosinase (TYR) and tyrosinase‐related protein‐1/2 (TRP‐1/2) expression. Melanin biosynthesis is initiated by tyrosine hydroxylation, catalyzed by TYR, to generate 3,4‐dihydroxyphenylalanine, which is then oxidized to dopaquinone through TYR‐mediated enzymatic reactions. TRP‐1/2 mediate subsequent oxidative modifications and finalize melanogenesis [6]. Newly synthesized melanin is packaged into melanosomes and transferred via dendritic processes to the adjacent keratinocytes. Consequently, skin pigmentation is predominantly determined by the dynamic equilibrium between melanosome biogenesis, melanin synthesis, intercellular transfer, and subsequent degradation [7]. Skin color is influenced by melanin and disturbed by other pigments, particularly lipofuscin. Lipofuscin, an aggregating complex of denatured proteins and peroxidized lipids, accumulates within the dysfunctional lysosomes. Lipofuscin progressively deposits in cells during cellular aging and exhibits broad‐spectrum light absorption across ultraviolet (UV) and visible wavelengths [8]. These photonic properties substantially reduce the light reflectance of the skin, leading to skin dullness and yellowness. Numerous studies have demonstrated a significant association between pigmentation and higher grades of sun exposure [9, 10, 11, 12]. Moreover, carbonylation and glycosylation processes in the skin are associated with UV radiation. However, sunscreen use cannot fully protect against UV‐induced damage. Therefore, to improve skin dullness in the Chinese population, it is crucial to address the pigmentation and yellowing caused by UV radiation while using sunscreens.

Currently, numerous studies have used complex formulations to improve skin dullness, which typically consists of multiple ingredients to achieve comprehensive, synergistic effects [13]. Nicotinamide, a component of coenzymes, including nicotinamide adenine dinucleotide (NAD+), NADH, NADP+, and NADPH [14], has been widely used as a raw material in various cosmetics. Nicotinamide exerts its whitening effects by attenuating UV‐induced DNA damage in epidermal melanocytes [15], inhibiting tyrosinase activity, and blocking melanosome transfer [16, 17, 18]. Tranexamic acid reduces melanin synthesis by inhibiting tyrosinase activity and is commonly used to treat melasma and other pigmentary skin disorders [19]. Notably, the combination of nicotinamide and tranexamic acid significantly improves facial pigmentation [20]. Moreover, hyaluronic acid‐coated tranexamic acid liposomes exhibit higher efficacy in treating local pigmentation compared to unmodified liposomes [21]. The combination of hyaluronic acid and nicotinamide effectively improves the clinical symptoms of skin aging [22]. Multiple clinical studies have demonstrated that the topical application of hyaluronic acid‐containing cosmeceuticals after various facial rejuvenation procedures enhances therapeutic efficacy and tolerance [23], further validating the dual role of hyaluronic acid in boosting efficacy and tolerability. Hydrolyzed red algae extract is rich in various bioactive compounds, among which polysaccharides and their degradation products strongly inhibit tyrosinase activity [24]. Although nicotinamide, tranexamic acid, hyaluronic acid, and hydrolyzed red algae exhibit anti‐melanogenic properties, the effects of their mixture on anti‐glycation and the reduction of lipofuscin deposition remain incompletely defined. In this study, we evaluated whether a mixture of hyaluronic acid, nicotinamide, hydrolyzed red algae, and tranexamic acid (HNHT) could effectively improve skin pigmentation and yellowing by reducing melanin deposition, skin glycation products, and lipofuscin accumulation.

## 2 Materials and Methods

### 2.1 Chemicals

α‐Melanocyte‐Stimulating Hormone (α‐MSH), dimethyl sulfoxide (DMSO), vitamin E (VE), 3‐(4,5‐Dimethylthiazol‐2‐yl)‐2,5‐diphenyltetrazolium bromide (MTT), melanin standard, and rapamycin were procured from Sigma‐Aldrich Chemical Co (St Louis, MO, USA). The anti‐melanoma glycoprotein 100 (GP100) antibody, microtubule‐associated proteins 1A/1B light chain 3B (LC3‐II) antibody, N‐carboxymethyl‐lysine (CML) antibody, Nrf2 antibody, and DAPI mounting medium were obtained from Abcam Inc. (Burlingame, CA, USA). KcGrowth and melanin model culture mediums were obtained from Guangdong Biocell Biotechnology Co. Ltd. (Guangzhou, China). The α‐MSH kit was obtained from Huamei Biotechnology (Wuhan, China). Glyoxal was bought from Adamas (Shanghai, China). The penicillin–streptomycin‐amphotericin B solution was obtained from Bioind (Kibbutz Beit HaEmek, Israel). Dulbecco's Phosphate‐Buffered Saline (DPBS) was obtained from Gibco (Thermo Fisher Scientific, Waltham, MA, USA). The hydrogen peroxide solution and aminoguanidine were supplied by Aladdin (Shanghai, China). Sudan Black B and Nuclear Fast Red staining solutions were acquired from Biosharp (Hefei, China). The RNA extraction kit was procured from Qiagen (Hilden, Germany). The reverse transcription and fluorescent quantitative polymerase chain reaction (PCR) kits were obtained from Takara (Shiga, Japan). Reactive oxygen species (ROS) fluorescent staining reagent was procured from CellRox Green (Invitrogen, Waltham, MA, USA). All other commercial‐grade reagents were obtained from the Sinopharm Group Co. Ltd. (Beijing, China). The HNHT formulation used in this study was a proprietary aqueous solution containing hyaluronic acid (0.1%, w/w), niacinamide (0.2%, w/w), hydrolyzed red algae extract (0.05%, w/w), and tranexamic acid (0.1%, w/w) in purified water. This stock solution was diluted with the appropriate culture medium or vehicle to achieve the final concentrations (such as 12.5%, 50%, or 100%, as specified in the method sections).

### 2.2 Cell Culture

Pigmented three‐dimensional (3D) skin equivalent models (MelaKutis, Guangdong Biocell Biotechnology Co. Ltd., Guangzhou, China) were cultured in a melanin model culture medium (Guangdong Biocell Biotechnology Co. Ltd., Guangzhou, China). Human epidermal keratinocytes (Guangdong Biocell Biotechnology Co. Ltd., Guangzhou, China) were cultured in medium KcGrowth (Guangdong Biocell Biotechnology Co. Ltd., Guangzhou, China). Melanocytes (Guangdong Biocell Biotechnology Co. Ltd., Guangzhou, China) were cultured in medium 254 (Thermo Fisher Scientific). Human primary dermal fibroblasts (Lifeline Cell Technology, Frederick, MD, USA) were cultured in the FibroLife S2 fibroblast medium (Lifeline Cell Technology, Frederick, MD, USA). Pigmented three‐dimensional (3D) skin equivalent models (MelaKutis, Guangdong Biocell Biotechnology Co. Ltd., Guangzhou, China) were cultured in a melanin model culture medium (Guangdong Biocell Biotechnology Co. Ltd., Guangzhou, China). The ex vivo skin model (Guangdong Biocell Biotechnology Co. Ltd., Guangzhou, China) was cultured using ex vivo skin culture medium (Guangdong Biocell Biotechnology Co. Ltd., Guangzhou, China). The cells were then incubated at 37°C with 5% CO_2_.

### 2.3 Cell Viability Determination

The MTT assay was used to assess cell viability. Human epidermal keratinocytes, human primary dermal fibroblasts, or melanocytes were grown in a 96‐well plate and incubated with HNHT or vehicle for 24 h. Next, 1 mL of 0.5 mg/mL MTT in phosphate‐buffered saline (PBS) was added to each well and incubated in the dark at 37°C for 4 h. After incubation, PBS‐washed cells were subjected to MTT colorimetric assay. Data derived from triplicate assays were analyzed.

To evaluate the HNHT effect on UVB radiation‐exposed keratinocyte viability, cells were incubated for 48 h, pretreated with HNHT or vehicle, exposed to UVB (50 mJ/cm^2^), and treated again before viability was assessed with MTT.

### 2.4 UVB‐Induced Skin Hyperpigmentation

#### 2.4.1 UVB‐Exposed MelaKutis and Evaluation of Appearance, Lightness, and Melanin Content and Distribution

The experimental procedure divided the models into four groups: Blank control (BC), negative control (NC), positive control (PC), and HNHT, each with six models. NC, PC, and HNHT received UVB treatment (50 mJ/cm^2^) daily, while BC received medium replacement daily. On days 3 and 5, the PC and HNHT groups were treated with 0.05% arbutin and 12.5% HNHT, respectively, through topical application. All models were cultured for 7 days.

A representative photograph was captured after model cultivation with the camera in manual mode: Focal length 5.8 mm, aperture f/8, shutter Speed 1/80s, and ISO 1600.

The L* value is a well‐known measure of skin color brightness. To measure the L* value, MelaKutis models were placed on a flat white surface with the cuticle upward and aligned in the detection hole of the colorimeter (Cortex Technology, SM II, Nordjylland, Hadsund, Denmark). Then, the L* value was recorded thrice.

After measuring the L* value, three MelaKutis models from each group were used for the melanin content analysis. The models were rinsed, lysed in sodium hydroxide with DMSO, and incubated, and melanin content was measured at 405 nm.

MelaKutis models from each group were reserved for melanin distribution. The models were fixed in a 4% paraformaldehyde solution for 24 h, embedded, sectioned, stained per the kit instructions, and photographed.

#### 2.4.2 UVB‐Exposed Keratinocytes

The experiment included four groups: BC, NC, PC, and HNHT, each with three replicates. BC And NC received 2 mL of culture medium, PC received 2 mL with 7 μg/mL VE, and HNHT received 2 mL of 12.5% HNHT. after treatment, the plates were incubated at 37°C with 5% CO_2_ for 24 h, and all but the BC were irradiated with UVB at 300 mJ/cm2. After irradiation, the plates were incubated for 24 h.

For ROS detection, the supernatant was discarded, the cells were incubated with an ROS‐sensitive dye for 1 h, and fluorescence was measured at 485 nm excitation and 520 nm emission.

#### 2.4.3 Melanosomes‐Feeding Keratinocytes

The experiment included there groups: NC, PC, and HNHT. For NC, 1 mL of fresh culture medium containing melanosomes was added to each well. PC received 1 mL of fresh culture medium containing melanosomes and 75 μM rapamycin/well. HNHT cells were treated with 1 mL of fresh culture medium containing melanosomes and 12.5% HNHT/well. Each group had three replicate wells, and the plates were incubated in an incubator at 37°C with 5% CO_2_ for 72 h.

### 2.5 Glyoxal‐Induced Skin Glycation

The experiment had four groups, BC, NC, PC, and HNHT, each with three replicates. Human dermal fibroblasts were seeded at 1.5 × 10^4^ cells/well in 24‐well plates and incubated for 48 h. The BC received no treatment, NC was treated with 0.5 mM glyoxal, PC with 0.5 mM glyoxal and 0.1 mM aminoguanidine, and HNHT with 0.5 mM glyoxal and 12.5% HNHT, followed by another 48 h of incubation.

The experiment had five groups, BC, NC, PC1, PC2, and HNHT, each with three replicates. Ex vivo skin explants were cultured in 6‐well plate chambers containing 3.7 mL of culture medium per well for 48 h with daily medium replacement. The NC, PC1, PC2, and HNHT groups received daily combined UV irradiation (UVA: 30 J/cm^2^; UVB: 50 mJ/cm^2^), while the BC group remained unirradiated. Post‐irradiation treatments were administered as follows: BC received fresh medium only; NC medium contained 3 mM methylglyoxal; PC1 medium contained methylglyoxal with vitamin C/E (VC: 100 μg/mL; VE: 7 μg/mL); PC2 medium contained methylglyoxal with 3 mM aminoguanidine sulfate; and the HNHT group received methylglyoxal‐containing medium plus topical 100% HNHT application. All samples were incubated at 37°C in 5% CO₂ for 24 h, repeating daily for 4 days, then maintained for 3 days without further irradiation or methylglyoxal.

### 2.6 H_2_O_2_ ‐Induced Skin Hyperpigmentation

The experiment included BC, NC, and HNHT groups, each with three replicates. Human keratinocytes were digested and seeded into chamber slide plates at 80%–90% confluence. After 48 h, the NC group received 2 mM H_2_O_2_, whereas the HNHT group received 2 mM H_2_O_2_ and 12.5% HNHT, followed by another 48 h of incubation.

### 2.7 Enzyme‐Linked Immunosorbent Assays

The quantification of α‐MSH in UVB‐exposed keratinocytes treated with HNHT was performed using ELISA kits following the manufacturer's instructions.

### 2.8 Quantitative Real‐Time Polymerase Chain Reaction (qRT‐PCR)

Total cellular RNA was isolated using an RNA extraction kit following the manufacturer's instructions. After reverse transcription into cDNA, related gene expression levels were detected using a fluorescence quantitative PCR instrument (LightCycler 480II, Roche, Mannheim, Germany). The results were analyzed and quantified using the 2^–ΔΔCt^ method to determine the gene expression levels of the cells in each group. The primer sequences used for the PCR tests are presented in Table S1.

### 2.9 Immunofluorescence Staining

Immunofluorescence staining was performed as described previously [25]. Briefly, the cells were rinsed with PBS, fixed with 4% paraformaldehyde for 30 min, blocked with 2.5% BSA for 1 h, and then incubated with primary antibodies against CML, GLO1, LC3II, GP100, and Nrf2 for 2 h at room temperature. After 1 h of secondary antibody incubation, the cells were mounted with DAPI, and fluorescence images were analyzed using ImageJ software.

For lipofuscin staining, keratinocytes were fixed with 4% paraformaldehyde at room temperature for 15 min. After rinsing the cells, Sudan Black B staining solution was added and incubated for 8 min. Cells were rinsed twice, followed by nuclear fast red staining for 10 min. Quantitative analysis was performed using the ImageJ software.

### 2.10 Statistical Analysis

All results are presented as mean ± standard deviation, and all statistical analyses were performed using the Statistical Package for the Social Sciences software (IBM Corporation; Armonk, NY, USA). For comparisons across multiple groups, a one‐way analysis of variance (ANOVA) was performed. If the ANOVA revealed a significant difference, Tukey's post hoc test was used to determine which groups differed. All statistical analyses were two‐tailed. A *p* < 0.05 was considered statistically significant, and a *p* < 0.01 was considered highly statistically significant.

## 3 Results

### 3.1 HNHT Reduced UVB‐Exposed Pigmentation in MelaKutis

**FIGURE 1:**
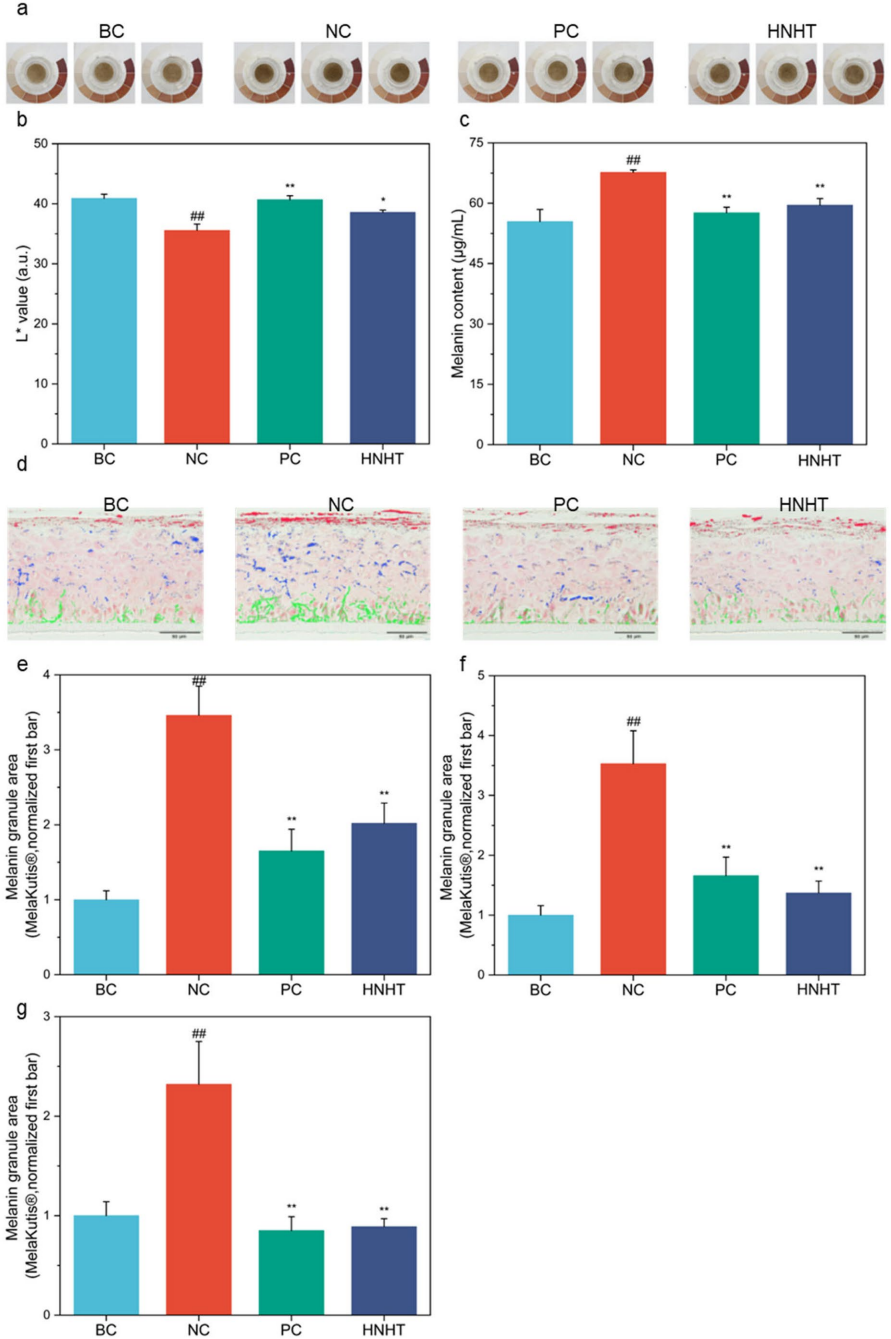
HNHT suppresses pigmentation in UVB‐exposed MelaKutis 3D skin models. (a) Dermoscopy of Melakutis. L* value (b) and total melanin content (c) of the Melakutis after application. (d) Melanin distribution of Melakutis. The scale bar equals 50 μm. Red illustrates the melanin particles within the stratum corneum, blue represents those in the upper layer (including spinous and granular layers), and green depicts the melanin particles located in the basal layer. (e–g) A detailed examination of melanin distribution across the stratum corneum (e), upper basal layer (f), and basal cell layer (g). BC: Blank control, NC: Negative control, PC: Positive control (0.05% kojic Acid). For comparisons with the BC group, #*p* < 0.05, and ##*p* < 0.01. For comparisons with the NC group, *p < 0.05, while **p‐value < 0.01.

To assess the efficacy of improving skin lightness and color (pigmentation), 0.05% arbutin and 12.5% HNHT were tested in a 3D skin equivalent mode model (Melakutis) subjected to UVB irradiation. UVB exposure induced significant darkening in this model, which was alleviated by arbutin and HNHT treatment (Figure 1a). Fontana‐Masson staining further confirmed elevated epidermal melanin content in UVB‐irradiated Melakutis. Fewer melanin deposits were observed in the HNHT‐treated samples (Figure 1d). This observation was supported by the melanin distribution in different epidermal layers of Melakutis (Figure 1e–g). The model demonstrated a significant decrease in melanin granules across the stratum corneum, upper basal layer, and basal cell layer compared to the NC group, with inhibition rates of 41.62%, 61.64%, and 61.19%, respectively (*p* < 0.05).

The L* value is a well‐known indicator of the overall tone. After UVB exposure, the L* value of Melakutis decreased significantly by 13.03% (Figure 1b). At a concentration of 12.5%, HNHT reversed the L* value decline induced by UVB by 8.44%. These results were consistent with the melanin quantification in MelaKutis (Figure 1c). UVB stimulated melanin production by 22.10% compared with BC. Moreover, 12.5% of HNHT reduced this level to 7.38%.

### 3.2 HNHT Reduced Melanin Production in Melanocytes

**FIGURE 2:**
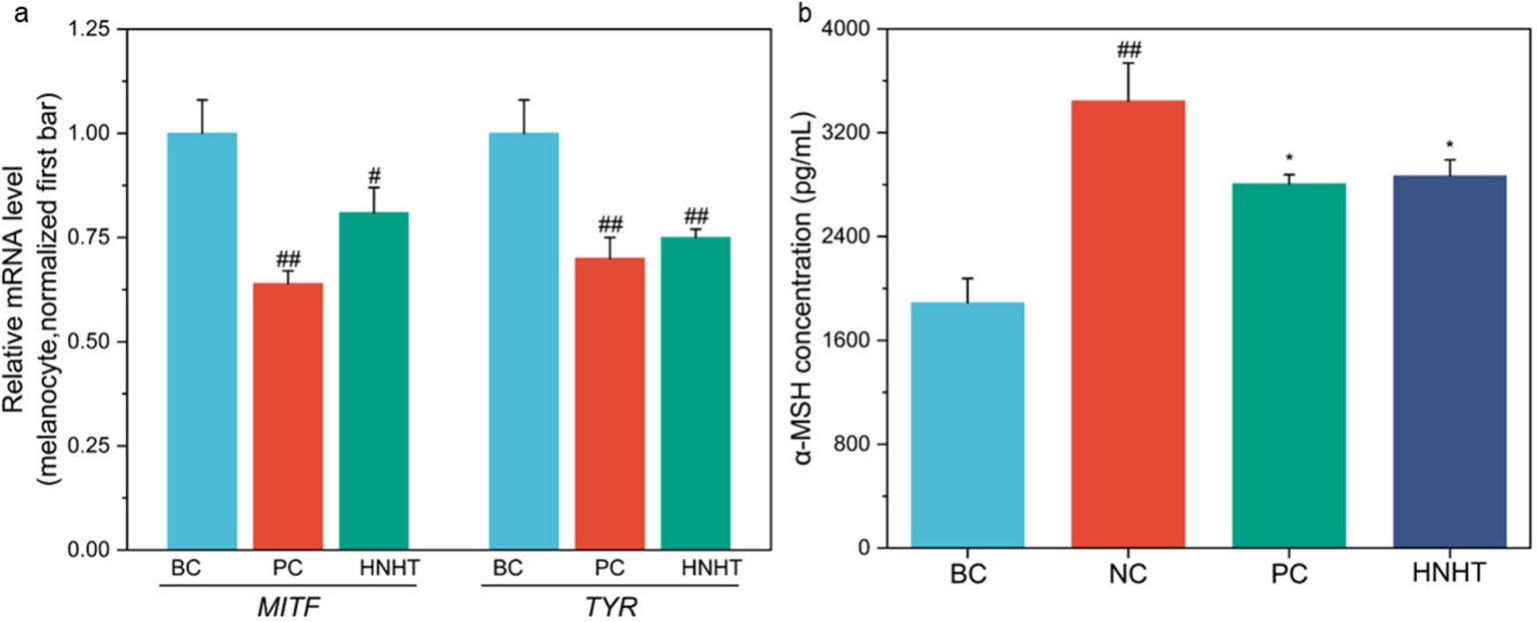
HNHT suppresses the expression of melanogenesis‐related genes in melanocytes (a) and α‐MSH secretion in UVB‐exposed keratinocytes (b). (a) Gene expression levels were quantified using the 2^–ΔΔCT^ method. For statistical analysis using the *t*‐test, the mRNA amplification fold changes in the BC group were normalized. BC: Blank control, NC: Negative control, PC: Positive control (a: 0.625 μg/mL glabridin; b: 7 μg/mL vitamin E). For comparisons with the BC group, #*p*‐value < 0.05, and ##*p*‐value < 0.01. For comparisons with the NC group, *p < 0.05, while **p < 0.01.

To evaluate the inhibitory effects of HNHT on melanogenesis‐related genes, glabridin was used as a positive control. Glabridin is a well‐characterized inhibitor that suppresses melanogenesis by inhibiting tyrosinase activity and downregulating the transcriptional levels of MITF signaling pathway‐related proteins with superior efficacy to α‐arbutin in in vitro and in vivo models [26]. Glabridin treatment resulted in significant reductions in the mRNA expression levels of *TYR* and *MITF* in melanocytes compared to BC (*p* < 0.001, Figure 2a). Similarly, 12.5% of HNHT administration induced a marked decrease in *TYR* and *MITF* mRNA expression, with expression levels reduced significantly compared to the BC (*p* < 0.001, Figure 2a). These results demonstrated that HNHT effectively inhibited melanogenesis in melanocytes. MC1R expressed on melanocytes is a critical positive melanogenesis modulator, which is instigated by keratinocyte‐released α‐MSH [6]. We evaluated the effect of HNHT on α‐MSH secretion in keratinocytes exposed to UVB irradiation (300 mJ/cm^2^). As determined by ELISA, UVB (300 mJ/cm^2^) exposure induced a 1.8‐fold increase in α‐MSH secretion from keratinocytes compared to BC (NC: 3444.72 ± 291.96 pg/mL versus BC: 1888.88 ± 187.93 pg/mL; *p* < 0.001; Figure 2b). Notably, co‐treatment with 12.5% HNHT significantly suppressed UVB‐triggered α‐MSH overproduction, reducing secretion levels to 2867.78 ± 122.88 pg/mL (*p* < 0.05, Figure 2b). Based on these data, HNHT inhibited melanogenesis under basal conditions and UVB stimulation by suppressing the melanocyte‐intrinsic expression of *TYR* and *MITF* and attenuating UVB‐triggered α‐MSH hypersecretion from keratinocytes.

### 3.3 HNHT Reduced Melanosome and Increased Autophagy in Melanosomes‐Feeding Keratinocytes

**FIGURE 3:**
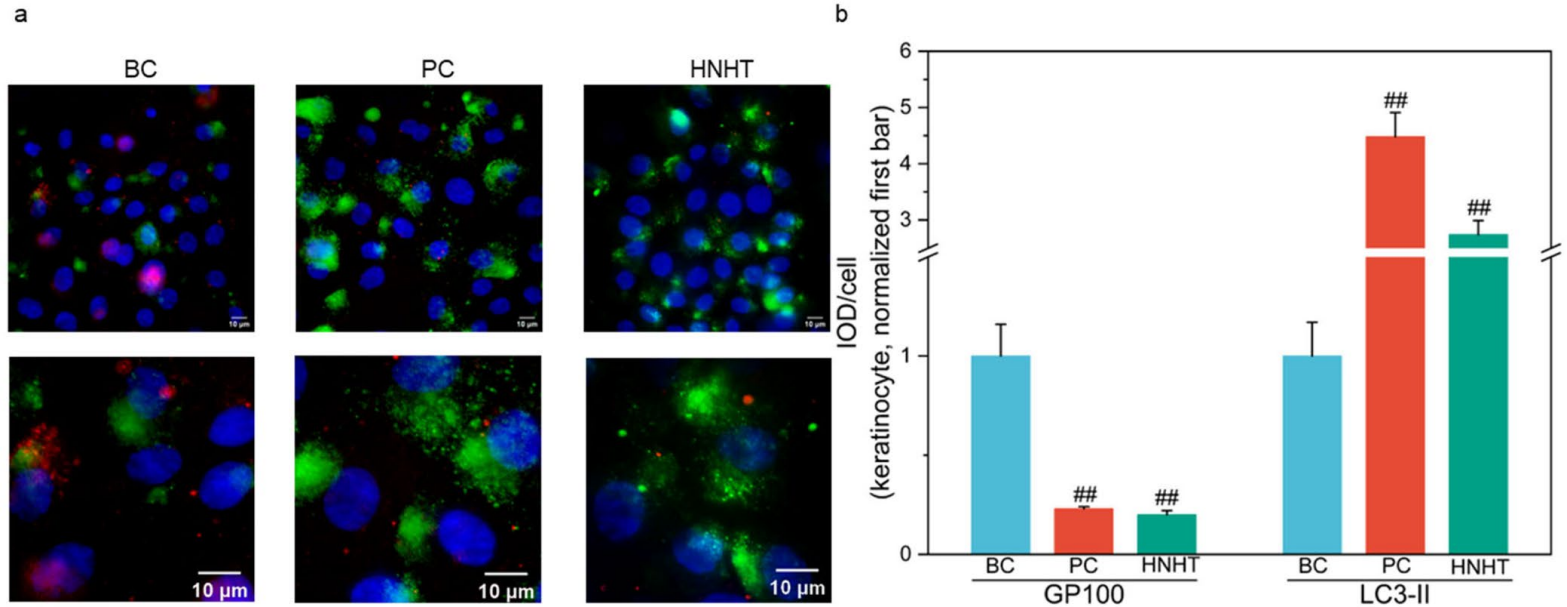
HNHT upregulates LC3‐II expression while downregulating GP100 levels in melanosomes‐feeding keratinocytes. (a) Super‐resolution microscopy (40×) images exhibiting LC3‐II (green), GP100 (red), and nuclei (DAPI, blue). (b) Quantitative analysis of fluorescence intensity expressed as IOD/cell. BC: Blank control, PC: Positive control (75 μM rapamycin). For comparisons with the BC group, #*p* < 0.05, and ##*p* < 0.01.

Keratinocytes participate in degrading melanin granules via the autophagic pathway, thereby influencing skin pigment metabolism [27]. Melanin is produced by melanocytes and stored in specialized organelles called melanosomes, which are subsequently transferred to adjacent keratinocytes through melanosomal transport [6]. As lysosome‐related organelles, melanosomes specifically express the transmembrane melanoma antigen protein GP100 on their membrane surface [28]. LC3‐II, a key component of autophagosomal membranes, is a direct marker of autophagosome biogenesis, with increased expression reflecting enhanced autophagosome formation [29]. The effect of HNHT on LC3‐II and GP100 was tested in melanosomes‐feeding keratinocytes. The immunofluorescence staining data revealed that rapamycin (positive control) validated the experimental model by markedly increasing LC3‐II levels (4.48 ± 0.31‐fold versus BC, *p* < 0.001) and reducing GP100 levels (0.23 ± 0.02‐fold versus BC, *p* < 0.001). Interestingly, at a concentration of 12.5%, HNHT significantly downregulated GP100 levels by 80% compared to BC and upregulated LC3‐II I levels by 174% (*p* < 0.01, Figure 3a,b). These findings indicate that HNHT promotes melanosome degradation by inducing autophagy in keratinocytes.

### 3.4 HNHT Enhanced the Antioxidant Capacity of UVB‐Exposed Keratinocytes

**FIGURE 4:**
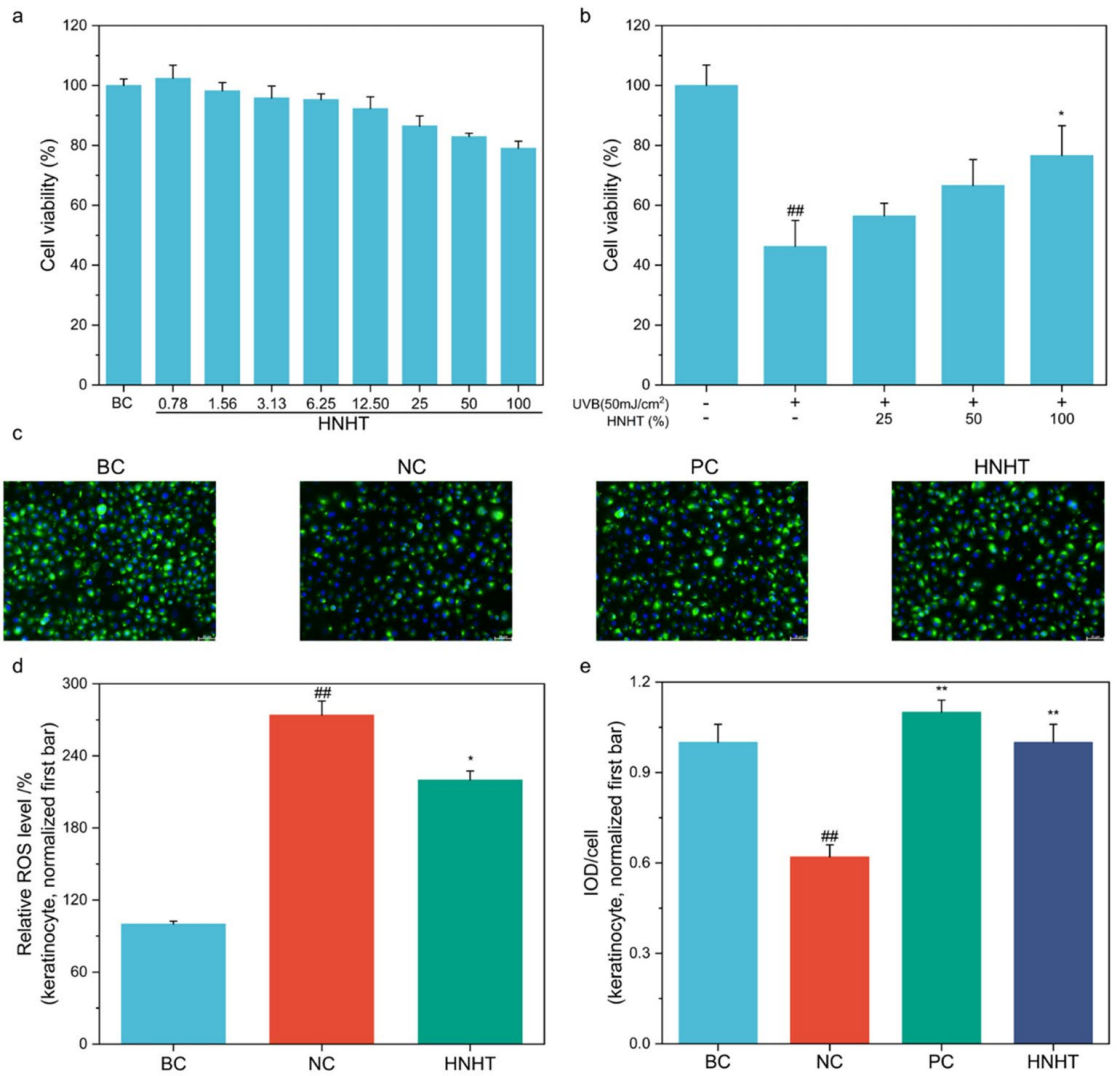
HNHT attenuates oxidative stress in UVB‐exposed keratinocytes. (a) Cell viability was determined by MTT assay in keratinocytes treated with varying HNHT concentrations (0.78%–100%) for 24 h without UVB irradiation. (b) Keratinocytes were irradiated with UVB (50 mJ/cm^2^) followed by treatment with HNHT (25%–100%) for 24 h. Cell viability was determined by MTT assay. (c) Nrf2 subcellular localization (green) was visualized by fluorescence microscopy (20× magnification) with DAPI nuclear counterstaining (blue), with quantitative analysis exhibited as IOD/cell (e). (d) Intracellular ROS levels were measured using DCFH‐DA fluorescence in HNHT‐treated keratinocytes with or without UVB irradiation (50 mJ/cm^2^). BC: Blank control, NC: Negative control, PC: Positive control (7 μg/mL vitamin E). For comparisons with the BC group, #*p* < 0.05, and ##*p* < 0.01. For comparisons with the NC group, *p < 0.05, while **p < 0.01.

The cytotoxic effect of HNHT on UVB‐irradiated keratinocytes was also investigated. MTT results demonstrated that compared to the untreated control, cell viability of keratinocytes was reduced to 79.04% after treatment with 100% HNHT (Figure 4a). Consequently, ≤ 100% HNHT was considered a nontoxic or sub‐cytotoxic HNHT concentration for further in vitro experiments in this investigation. As presented in Figure 4b, UVB exposure reduced cell viability to 46.3% compared to the control group. Pre‐application of 50% HNHT increased the cell viability to 66.62%. UVB irradiation triggers ROS generation in keratinocytes. Therefore, we tested whether HNHT could protect cells from UVB radiation‐induced intracellular ROS production. DCFH‐DA fluorescence results revealed that UVB (50 mJ/cm^2^) alone exposed keratinocytes significantly upregulated (more than 2.7‐fold) intracellular ROS levels. Nevertheless, pretreatment with 50% HNHT significantly ameliorated this effect (Figure 4d), indicating the antioxidant activity of HNHT. Antioxidant Nrf2 is a critical cytoprotective pathway that protects skin cells from ROS and electrophilic insults caused by UVB exposure. During the cytoprotective pathway, Nrf2 separates from Keap‐1 and translocates to the cellular nucleus to express antioxidant genes. Immunofluorescence data revealed that 12.5% HNHT facilitated the Nrf2 level in UVB‐exposed keratinocytes (Figure 4c,e), signifying the antioxidant efficacy of HNHT.

### 3.5 HNNT Enhanced Gene Expression for AGE Clearance and Reduced CML Formation

Skin glycation, a primary factor contributing to skin yellowing, generates advanced glycosylation end products (AGEs) that induce pathological alterations [4, 5]. CML is a key biomarker for evaluating skin glycation levels. Glyoxalase 1 (GLO1), glyoxalase 2 (GLO2), and glutathione peroxidase 1 (GPX1) form a synergistic network in the anti‐glycation process [29]. The glyoxalase system (GLO1/GLO2) directly reduces AGE production by metabolizing glycolytic byproducts (e.g., methylglyoxal) to block non‐enzymatic glycation reactions with proteins. As an antioxidant enzyme, GPX1 inhibits oxidative stress‐mediated AGE formation by scavenging free radicals and supports GLO1/GLO2 activity by maintaining glutathione levels. Collectively, these three components block AGE generation and toxicity through two integrated mechanisms: Metabolic regulation of glycation intermediates and antioxidant defense against oxidative stress.

**FIGURE 5:**
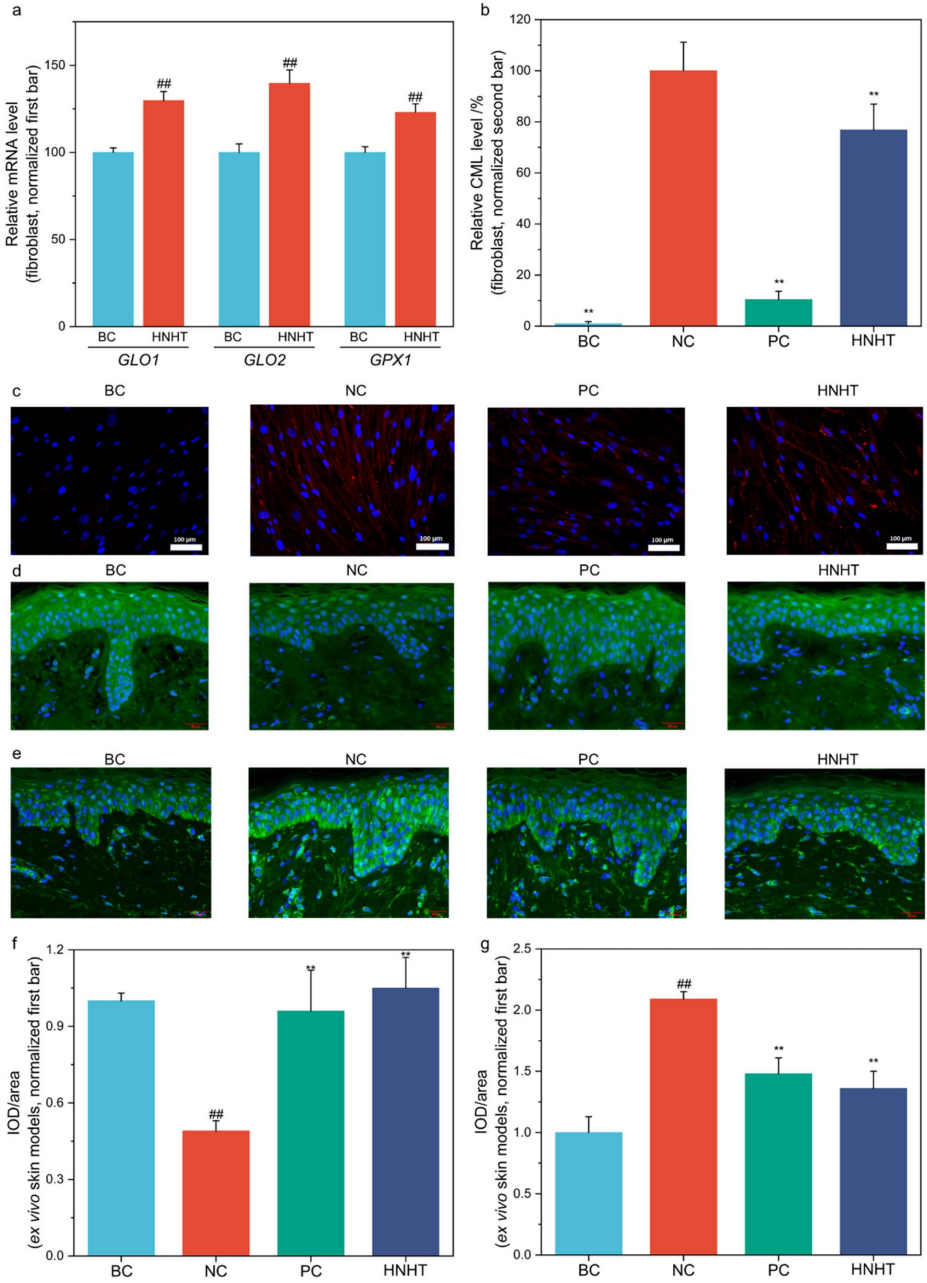
HNHT enhances glycation clearance gene expression and suppresses CML formation in glycation‐induced fibroblasts and ex vivo skin models. (a) Expression of glycation clearance‐related genes in fibroblasts was analyzed by RT‐qPCR after 50% HNHT treatment. (b) CML levels in glyoxal‐induced fibroblasts were quantified by immunofluorescence staining. (c) Immunofluorescence images (10× magnification) of glyoxal‐exposed fibroblasts exhibiting CML (red) and DAPI‐stained nuclei (blue). (d and f) GLO1 expression in methylglyoxal+UVA + UVB‐induced 3D skin models: (d) Representative immunofluorescence images (20×) of GLO1 (green) and nuclei (DAPI, blue); (f) Quantitative analysis of GLO1 expression. (e and g) CML levels in methylglyoxal+UVA + UVB‐induced ex vivo skin models: (e) Immunofluorescence images (20×) of CML (green) and nuclei (DAPI, blue); (g) Quantitative analysis of CML expression. BC: Blank control, NC: Negative control, PC: Positive control (b‐c: 0.1 mM aminoguanidine sulfate; d–g: 3 mM aminoguanidine sulfate). For comparisons with the BC group, #a *p* < 0.05, and ##a *p* < 0.01. For comparisons with the NC group, *p < 0.05, while **p < 0.01.

In fibroblasts and ex vivo skin models, differential expression patterns of the skin glycation marker (CML) and anti‐glycation‐associated enzymes were determined after treatment with HNHT. RT‐PCR results revealed that 50% of HNHT significantly upregulated the gene expression of *GLO1*, *GLO2*, and *GPX1* in fibroblasts (Figure 5a). Additionally, immunofluorescence staining revealed that glyoxal significantly induced CML expression in fibroblasts (*p* < 0.01), while treatment with aminoguanidine sulfate (positive control for anti‐glycation) significantly suppressed CML expression (*p* < 0.01, Figure 5b), confirming the validity of our experimental model. Treatment with 50% HNHT resulted in a significant 23.16% reduction in CML expression compared to the glyoxal‐induced group (*p* < 0.01, Figure 5b,c). This result was also confirmed in an ex vivo skin model. Immunofluorescence data revealed that combined stimulation with methylglyoxal (3 mM) and UV (UVA: 30 J/cm^2^ + UVB: 50 mJ/cm^2^) radiation in the ex vivo skin model led to a 51% reduction in GLO1 levels and a > 2‐fold increase in CML expression. However, treatment with 100% HNHT increased GLO1 levels by 114.3% (from 0.49 to 1.05 IOD/area; Figure 5d,f) and decreased CML levels by 34.93% (from 2.09 to 1.36 IOD/area; Figure 5e,g) in the ex vivo skin model, indicating the anti‐skin glycation capacity of HNHT.

### 3.6 HNHT Inhibited Lipofuscin Formation in H_2_O_2_ ‐Induced Keratinocytes

It has been demonstrated that UV irradiation increases oxidative protein damage and melanogenesis and directly promotes lipofuscin accumulation in the skin, leading to solar lentigines [30]. Aging is associated with a significant increase in skin lipofuscin content, which disrupts cellular homeostasis by reducing proteasomal activity, thereby exacerbating aging‐related hyperpigmentation [31]. In a skin explant model simulating senile lentigo, researchers successfully established a pathological model of lipofuscin accumulation by inducing oxidative stress through systemic treatment with H_2_O_2_ [30]. Accordingly, 2 mM H_2_O_2_ was applied to induce lipofuscin formation in human primary keratinocytes, resulting in a significant 39.6% increase in lipofuscin production. Subsequent treatment with 50% HNHT significantly reduced lipofuscin levels to 47.8% of the H_2_O_2_‐induced control levels, corresponding to an inhibition rate of 52.20%. These results demonstrate that 50% HNHT suppresses H_2_O_2_‐induced lipofuscinogenesis in human primary epidermal keratinocytes.

## 4 Discussion

Skin dullness, which is characterized by uneven pigmentation and yellowish discoloration, is a globally prevalent cosmetic concern. A 2017 consumer study by Procter & Gamble revealed that over 40% of women worldwide report skin dullness as a significant issue, with the prevalence being particularly high in China, where more than 60% of women experience this concern [32]. This condition arises from multifaceted factors, including excessive melanin production, accumulation of AGEs, and lipofuscin deposition. Although existing therapies often target melanogenesis or glycation individually, their limited efficacy underscores the need for a multitargeted approach. In this study, we evaluated a novel HNHT formulation (hyaluronic acid, niacinamide, hydrolyzed red algae, and tranexamic acid) for its ability to simultaneously inhibit melanin synthesis, reduce glycation‐induced damage, and suppress lipofuscin accumulation. Our results demonstrate that HNHT significantly improves skin brightness, reduces melanin content, downregulates key melanogenic genes (*TYR* and *MITF*), enhances melanosome degradation via autophagy, and diminishes AGEs and lipofuscin deposition, highlighting its potential as a comprehensive anti‐dullness therapy.

### 4.1 HNHT Inhibits Melanogenesis via Multiple Pathways

UVB radiation is a major extrinsic factor that drives melanin synthesis by upregulating α‐MSH secretion from keratinocytes and activating the MC1R‐MITF‐TYR axis in melanocytes [6]. Our findings reveal that HNHT significantly suppresses UVB‐induced α‐MSH overproduction in keratinocytes (Figure 2b), thereby attenuating MC1R‐mediated melanogenic signaling. Furthermore, HNHT downregulates key melanogenic genes (*TYR* and *MITF*) (Figure 2a), consistent with previous reports that tranexamic acid modulates melanogenesis by suppressing tyrosinase activity, reducing TRP1/TRP2 expression and lowering MITF protein levels [33]. HNHT also enhances melanin degradation by promoting autophagy in keratinocytes, as evidenced by increased LC3‐II (an autophagosome marker) and decreased GP100 (a melanosome marker) (Figure 3a,b). Intriguingly, while existing studies confirm that tranexamic acid does not induce autophagy in keratinocytes to facilitate melanin degradation [34], it suppresses melanogenesis by activating the autophagic system in melanoma cells [33]. Furthermore, a nicotinamide‐optimized low‐pH formulation upregulates mRNA and protein levels of ATG5 [35], a critical autophagy‐related gene, in human epidermal keratinocytes. Clinically, the combined topical application of nicotinamide and tranexamic acid has demonstrated efficacy in reducing facial hyperpigmentation [20], partly through inhibiting melanocyte dendricity‐associated ligands within pigmented lesions [36]. Based on these findings, we hypothesize that HNHT‐mediated suppression of melanosome accumulation in cocultured keratinocytes may involve autophagy‐dependent mechanisms, potentially attributable to the synergistic effects of nicotinamide and tranexamic acid. However, further validation is required to delineate their individual contributions.

### 4.2 HNHT Mitigates Skin Glycation and AGE Accumulation

Recent studies highlight the pivotal role of AGEs in skin dullness and yellowing, particularly in young populations. A clinical study by Laughlin et al. [32] demonstrated that epidermal AGE accumulation correlates strongly with dull skin appearance, even among individuals aged 20–29. Immunofluorescence staining of facial biopsies revealed significantly higher levels of CML in the epidermis of subjects with dull skin compared to non‐dull counterparts. In the context of non‐enzymatic skin glycation, GLO1, GLO2, and GPX1 form a synergistic defensive network against glycation [29]. The glyoxalase system (GLO1/GLO2) inhibits AGE generation by eliminating methylglyoxal, a highly reactive glycation intermediate. GPX1 indirectly supports the glyoxalase function by maintaining glutathione homeostasis via its antioxidant activity. Our data demonstrate that HNHT upregulates *GLO1*, *GLO2*, and *GPX1* (Figure 5a), enhancing the degradation of methylglyoxal, a key AGE precursor. Consequently, HNHT reduces CML formation by 23.16% in fibroblasts (Figure 5b,c) and 34.93% in the ex vivo skin model (Figure 5d–g), comparable to the anti‐glycation effects of aminoguanidine. These results align with studies revealing that nicotinamide boosts NAD + ‐dependent glyoxalase activity [14]. However, hyaluronic acid improves skin hydration and barrier function, indirectly reducing glycation stress [23].

### 4.3 HNHT Reduces Lipofuscin Deposition

**FIGURE 6:**
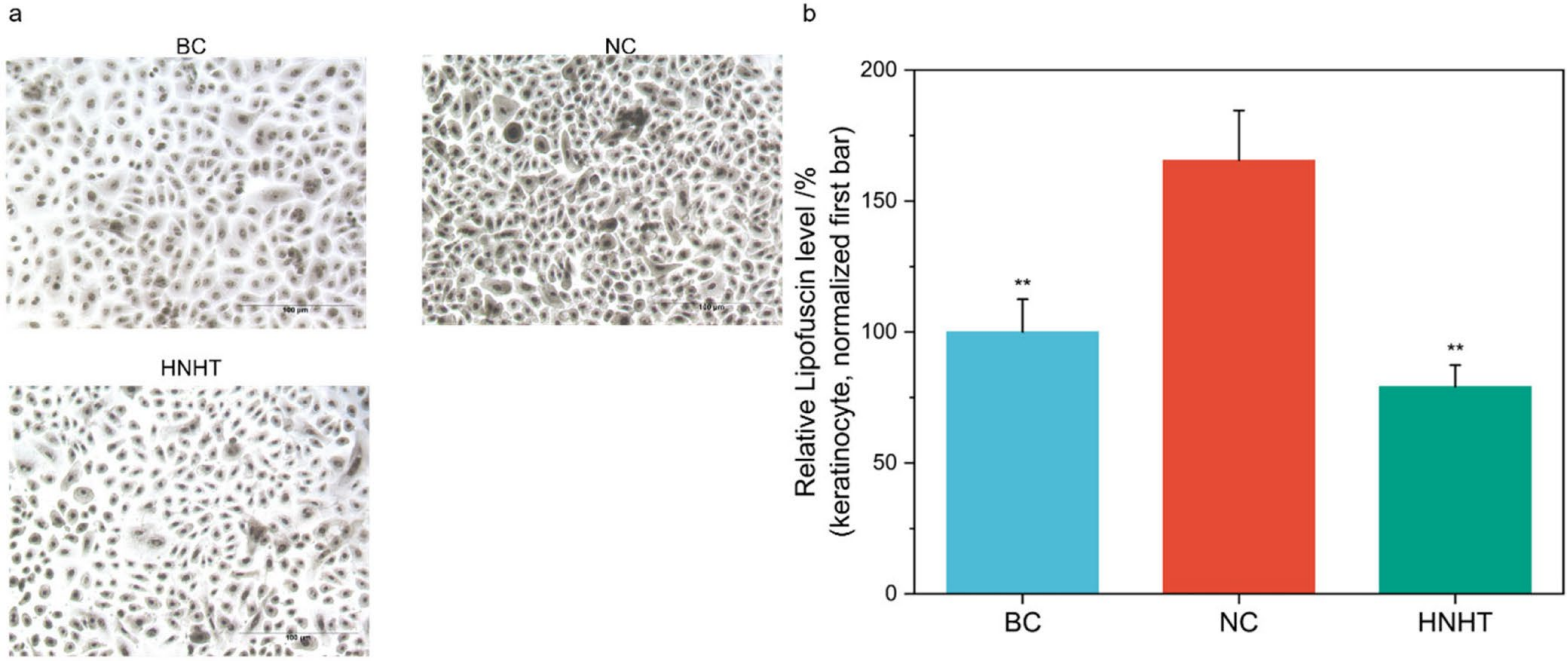
HNHT inhibits H_2_O_2_‐induced lipofuscin accumulation in fibroblasts. Fibroblasts were first exposed to H_2_O_2_, followed by treatment with or without 50% HNHT. (a) Representative immunohistochemical images of lipofuscin deposition in fibroblasts. (b) Quantitative analysis of lipofuscin staining intensity, expressed as a percentage relative to NC (set as 100%). BC, blank control; NC, negative control. For comparisons with the NC group, *p < 0.05, while **p < 0.01.

Lipofuscin, a fluorescent pigment composed of cross‐linked protein‐lipid aggregates, accumulates in aged skin due to lysosomal dysfunction and oxidative stress. Its broad‐spectrum light absorption properties significantly impair skin reflectance, contributing to the characteristic dull and sallow appearance of aging skin [8]. Our study demonstrates that HNHT suppresses H_2_O_2_‐induced lipofuscin formation by 52.20% (Figure 6), likely through its antioxidant properties. Nicotinamide exerts significant inhibitory effects on photosensitization‐induced lipid peroxidation through scavenging singlet oxygen (^1^O_2_) and partial reactive oxygen species (e.g., superoxide anion, O_2_^−^) [37]. Meanwhile, hyaluronic acid demonstrates potent antioxidative capacity in mitigating oxidative stress associated with skin aging [38].

Moreover, the HNHT formulation protects keratinocytes from UVB‐induced damage by reducing ROS generation and upregulating Nrf2 expression. As a key transcriptional regulator, Nrf2 activates antioxidant response elements (AREs) in the promoter regions of cytoprotective genes, thereby enhancing the antioxidant enzyme expression. The HNHT formulation demonstrates a comprehensive anti‐dullness mechanism through synergistic modulation of three interconnected pathological pathways: (1) Inhibition of lipofuscin deposition, (2) suppression of glycation through GLO system regulation, and (3) attenuation of melanogenesis. This multitargeted approach effectively addresses the yellowish discoloration (resulting from AGE accumulation and lipofuscin deposition) and hyperpigmentation (caused by excessive melanin production) that collectively contribute to skin dullness. The coordinated action on these pathways stems from their shared oxidative stress pathogenesis, where HNHT's Nrf2‐ARE activation provides a unifying mechanism that complements its specific anti‐glycation and anti‐melanogenic effects. Our results demonstrate that HNHT treatment induces significant whitening effects in UVB‐irradiated MelaKutis, as evidenced by a marked improvement in skin lightness (L* value), with an 8.44% increase compared to untreated controls.

### 4.4 Study Strengths and Limitations

This study presents several notable advances in skin dullness research. First, this is the first comprehensive investigation to simultaneously target the three key pathological factors of skin dullness: Melanin overproduction, glycation, and lipofuscin deposition using a combinatorial approach. Second, the HNHT formulation demonstrates synergistic efficacy by integrating multiple active ingredients (hyaluronic acid, niacinamide, hydrolyzed red algae, and tranexamic acid) that act through distinct yet complementary mechanisms. Third, our study provides pioneering evidence for the anti‐glycation and anti‐lipofuscin effects of this combination, establishing a new potential approach for treating age‐related skin discoloration.

Although this study offers valuable insights, some limitations should be addressed. First, the research focused exclusively on the combined effects of HNHT's four components at a fixed ratio, without evaluating the individual contributions of hyaluronic acid, niacinamide, hydrolyzed red algae, or tranexamic acid. This leaves unanswered questions about the relative importance of each ingredient in the observed effects. Second, while we identified HNHT's activity across three key pathways, the mechanistic details, particularly regarding melanogenesis inhibition and anti‐glycation pathways, were not fully elucidated. More in‐depth investigations into the molecular targets and signaling cascades would strengthen our mechanistic understanding. Future studies should address these gaps through component‐specific experiments and more comprehensive pathway analyses. Despite these limitations, our findings establish HNHT as a promising multi‐mechanistic formulation for skin dullness.

## 5 Conclusion

This study demonstrates that the HNHT formulation (hyaluronic acid, niacinamide, hydrolyzed red algae, and tranexamic acid) effectively improves skin dullness through a multitargeted mechanism involving melanogenesis inhibition (via α‐MSH/MC1R/MITF signaling suppression and enhanced melanosome autophagy), anti‐glycation activity (through upregulation of *GLO1*/*GLO2* and *GPX1* leading to reduced AGE accumulation), and decreased lipofuscin deposition. The combined action of these pathways results in significant improvements in skin brightness (L* value) and overall complexion, highlighting the potential of HNHT as a comprehensive therapeutic approach for addressing the multifactorial causes of skin dullness, particularly in populations prone to hyperpigmentation and glycation‐related discoloration.

## Author Contributions

Z.W. and P.L. designed this study. Z.W. and Y.F. conducted the experiments, performed statistical analyses, and interpreted the experimental data. Z.W. drafted the manuscript. All authors including Z.W., Y.F., and P.L. participated in data analysis through constructive discussions and critically reviewed the manuscript. All authors read and approved the final manuscript.

## Funding

The authors have nothing to report.

## Conflicts of Interest

The authors declare no conflicts of interest.

## Supporting information


**Table S1:** Primers used in this study.

## Data Availability

The data that support the findings of this study are available from the corresponding author upon reasonable request.
